# Physiological Response Mechanisms of *Triplophysa strauchii* Under Salinity Stress

**DOI:** 10.3390/biology14091202

**Published:** 2025-09-05

**Authors:** Shixin Gao, Jinqiu Wang, Kaipeng Zhang, Guanping Xing, Yunhong Tan, Lulu Chen, Tao Ai, Shijing Zhang, Yumeng Chen, Zhulan Nie, Jie Wei

**Affiliations:** 1 College of Life Science and Technology, Tarim University, Alar 843300, China; follow_spot@163.com (S.G.); bestboy_qiu@163.com (J.W.); zkp19980604@163.com (K.Z.); xingguanping1111@163.com (G.X.); tyh3429@163.com (Y.T.); chenlulurc@163.com (L.C.); 2 Xinjiang Production & Construction Corps Key Laboratory of Protection and Utilization of Biological Resources in Tarim Basin, Alar 843300, China; 3 Xinjiang Production and Construction Corps Aquaculture Technology Promotion General Station, Urumqi 830002, China; taoai202506@163.com; 4 Xinjiang Yutian County Fengze Technology Aquaculture Co., Ltd., Hotan 848400, China; 17797917561@163.com; 5 Xinjiang Production and Construction Corps 9th Division Animal Husbandry and Aquaculture Development Center, Tacheng 834300, China; 15276870775@163.com

**Keywords:** *Triplophysa strauchii*, salinity tolerance threshold, osmoregulation, antioxidant parameters

## Abstract

Salinity, a critical environmental factor for fish survival, remains poorly understood in terms of how *Triplophysa strauchii*, a characteristic fish in Northwest China, physiologically responds to salinity stress. This study aimed to determine its salinity tolerance threshold and explore the associated physiological damage mechanisms. Six salinity gradients (11, 11.7, 12.5, 13.3, 14.3, 15.1 ppt) and a freshwater control group were established. Acute toxicity tests recorded mortality and behavior, while physiological–biochemical assays measured ion concentrations and enzyme activities in gills, kidneys, liver, intestines, and plasma over 96 h. The results showed a 96-hour median lethal concentration of 13.31 ppt and a safe concentration of 4.05 ppt. Gills and kidneys, as primary osmoregulatory organs, responded rapidly, whereas the liver and intestine lagged. Salinity ≤ 13.3 ppt allowed the fish to maintain homeostasis via physiological adjustments, but ≥14.3 ppt caused ion imbalance, immune function was significantly suppressed, and irreversible damage. These findings clarify the species’ salinity adaptation strategies, providing a basis for further research on chronic salinity stress.

## Simple Summary

Rivers in northwest China have changing salt levels, which threaten a local fish called *Triplophysa strauchii*. As such, we studied them to find out how much salt they can take and how their bodies react. We tested six different salt levels and a freshwater group, watching how many fish survived, how they acted, and how their bodies worked over four days. We found that half of the fish died when the salt level reached 13.31 ppt, and a safe level was 4.05 ppt. Their gills and kidneys quickly adjust to control water and salt, but their liver and intestines react more slowly. If salt levels stay below 13.31 ppt, the fish can stay healthy. But above 14.3 ppt, their bodies get out of balance, their ability to fight off harm stops working, and they get irreversible physiological dysfunction. This helps us protect these fish by controlling river salt levels, especially in dry areas where salt builds up, keeping their populations safe.

## 1. Introduction

Salinity, a pivotal environmental factor in aquatic systems, influences all stages of fish life cycles, from embryonic development to adult reproduction [1,2]. It modulates critical processes such as egg buoyancy regulation, maintenance of osmotic balance, and migration route selection, all of which depend on appropriate salinity gradients [3,4,5].

Fish have evolved a coordinated salinity regulation system centered on the gills, kidneys, liver, and intestines, which maintain homeostasis through structural and functional synergies [6,7]. Gills: As the primary interface with external water, gills regulate electrolyte balance via transport proteins like Na^+^/K^+^-ATPase(Adenosine Triphosphate) in gill filament epithelial cells. Hypertonic stress (e.g., increased ambient salinity) causes gill cell swelling, reducing ion transport efficiency and disrupting electrolyte balance [8,9]. Kidneys: These organs fine-tune urine production to maintain fluid homeostasis. In high-salinity environments, renal tubules enhance water reabsorption, but prolonged stress induces pathological changes (e.g., tubular epithelial vacuolization, interstitial fibrosis), impairing renal function and fluid osmotic regulation [10,11]. Liver: Initially, hepatocytes boost lysosomal activity to clear pathogens and debris and accelerate IgM (Immunoglobulin M) synthesis for immune defense at mucosal barriers (e.g., gills, skin). However, prolonged hypertonic exposure damages mitochondria, reducing lysosomal efficiency and increasing infection risk [12,13]. Intestines: As a key digestive and absorptive organ, the intestine maintains electrolyte and osmotic balance. Elevated ambient salinity drives water efflux from the intestinal lumen, causing epithelial cell dehydration and shrinkage, which disrupts ion transport and compromises balance [14,15].

Salinity fluctuations trigger oxidative and immune responses in fish. Drastic salinity changes induce abnormal mitochondrial respiration, generating excessive reactive oxygen species (ROS) like superoxide anions and hydrogen peroxide [16,17]. Concurrently, immune function is impaired, with reduced IgM levels in the liver and plasma and decreased lysozyme activity [18,19]. Sustained salinity stress can thus lead to tissue damage via ion imbalance, osmoregulatory dysfunction, and energy metabolism abnormalities, potentially causing irreversible harm or population decline.

The Emin River basin, a typical arid inland system, has a 7:1 precipitation–evaporation ratio (271 mm annual precipitation vs. 2200 mm evaporation). Coupled with weathering of Tertiary saline strata (e.g., gypsum, halite), this creates a natural salinity gradient (1.2–35 ppt) in terminal lakes, making it ideal for studying arid-region salinization [20]. Recent decades have seen rising salinity (NaCl contributing > 70%), with some tributaries reaching 10–14 ppt [21,22]. Anthropogenic factors (reservoir operations, agricultural return flow) and reduced glacial meltwater (due to climate warming) further elevate downstream salinity [23,24], exacerbated by declining annual runoff (1980–2007) [25].

*Triplophysa strauchii*, a key indicator species in the Emin River Basin, maintains alpine cold-water food web stability but has declined sharply, being listed as “Near Threatened” [26]. Existing research focuses on its ecology and diversity, leaving gaps in understanding physiological responses to salinity stress. This study aimed to achieve the following: Ⅰ. determine the 96-hour median lethal concentration (LC_50_) and safe concentration (SC); Ⅱ. elucidate the dynamic response patterns of osmoregulation, antioxidant defense, and immune systems; and Ⅲ. reveal the pathological damage mechanisms in gill, liver, intestine, and kidney tissues, thereby providing theoretical insights into fish injuries caused by salinization.

## 2. Materials and Methods

### 2.1. Fish

**Figure 1:**
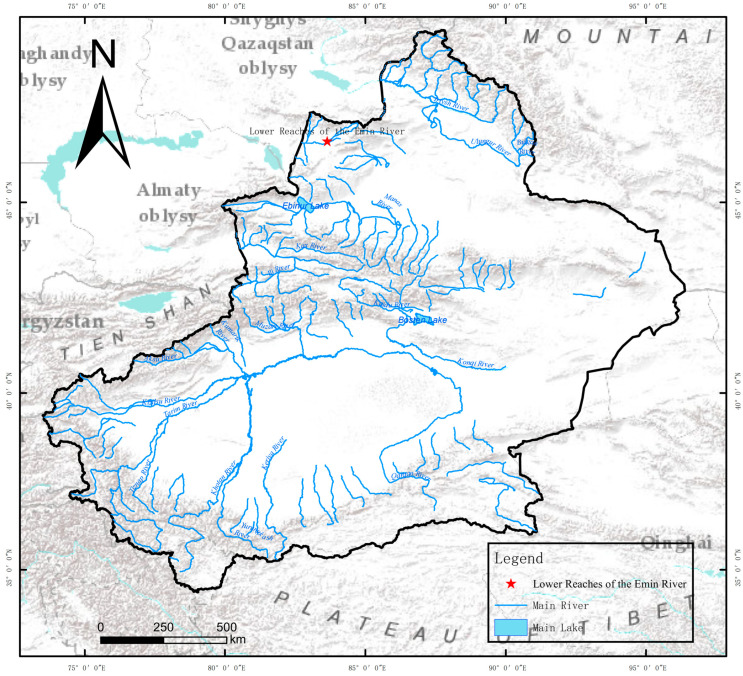
Schematic diagram of sampling point distribution. ☆: sampling point.

A total of 650 wild *Triplophysa strauchii* were collected from the Emin River system in Xinjiang (Figure 1), with a body mass of 11 ± 0.53 g and body length of 11.5 ± 0.61 cm. The fish were acclimated in tap water aerated for over 48 h in the laboratory for 1 week under conditions of water temperature (21 ± 1) °C, pH (7.78 ± 0.09), dissolved oxygen (9.00 ± 1.07) mg/L, and salinity (0.645 ± 0.37) ppt, which complied with the Fishery Water Quality Standard (GB 11607-89) [27]. Feeding was withheld 24 h prior to the experiment. The aquariums used measured 60 cm × 48 cm × 38 cm, and the experimental reagent NaCl (analytical reagent grade, AR ≥ 99.5%) was supplied by Nanjing Chemical Reagent Co., Ltd., Nanjing, China. All experiments were conducted in accordance with the guidelines issued by the Laboratory Animal Center of Tarim University. The protocol was approved by the Animal Research Ethics Committee of Tarim University (approval no. PB20250521002).

### 2.2. Experimental Design

Concentration gradients were set based on preliminary experiment results (preliminary experiments showed no mortality below 11 ppt and 100% mortality within 24 h above 16 ppt, thus 11–16 ppt was selected), six salinity gradients were set: 11 ppt, 11.7 ppt, 12.5 ppt, 13.3 ppt, 14.3 ppt, and 15.1 ppt, with a freshwater control group at 0.7 ppt. The experiment comprised two parts. (1) Acute toxicity test: Each salinity group included 36 fish divided into 3 parallel groups (12 fish/group). Mortality was recorded and behavioral changes were observed at 0, 6, 12, 24, 48, 72, and 96 h. (2) Physiological and biochemical experiments: Each salinity group contained 54 fish in 3 parallel groups (18 fish/group). Samples (gills, liver, intestine, kidney, and plasma) were collected at 0, 6, 12, 24, 48, 72, and 96 h. Throughout the experiment, conditions were maintained as follows: photoperiod 13 L:11 D, no feeding, micro-aeration (≥6 mg/L). One-third of the water was replaced daily using siphoning under dim light to collect feces. After water renewal, salinity was re-measured to ensure concentration stability. Salinity was determined using a Leici ZD-2 salinometer (Shanghai Yidian Scientific Instruments Co., Ltd., Shanghai, China), stabilized for 24 h after preparation, and re-confirmed after impurity removal.

In the acute toxicity test group, continuous observations were conducted over 96 h. Mortality was recorded at 0, 6, 24, 48, 72, and 96 h, alongside behavioral responses at each time point. Death was defined as the fish sinking to the bottom without movement, gill cover cessation, and no tail response to glass rod stimulation.

### 2.3. Plasma and Tissue Sample Collection

In the physiological and biochemical group, 9 fish were randomly sampled from each parallel group at each time point (0, 6, 12, 24, 48, 72, 96 h), with a total of 9 fish per time point (3 groups × 3 fish). Fish were anesthetized with 200 mg/L MS-222 (Hangzhou Animal Pharmaceutical (Hangzhou) Co., Ltd., Hangzhou, China), and blood was collected using heparinized syringes. For the first 3 fish, plasma (separated by centrifugation) and tissues (gill, liver, intestine, kidney) were harvested, snap-frozen in liquid nitrogen, and stored for enzyme activity and ion concentration assays.

### 2.4. Enzyme Activity Assays

Tissue samples: Tissues were homogenized in liquid nitrogen-cooled mortars with 9 volumes of ice-cold saline (*w*/*v* = 1:9). Homogenates were centrifuged (Eppendorf 5810R, (Origin/Brand: Germany Eppendorf, Hamburg, Germany)) at 3000 rpm for 10 min at 4 °C; supernatants were diluted 5-fold with saline, and 50 μL of the diluted supernatant was used for assays.

Plasma samples: Plasma was obtained by centrifuging blood at 3000 rpm for 10 min at 4 °C. Activities of IgM, acid phosphatase (ACP) (A060-1-), alkaline phosphatase (AKP) (A059-1-1), superoxide dismutase (SOD) (A001-3-2), catalase (CAT) (A007-1-1), malondialdehyde (MDA) (A003-1-1), and Na^+^/K^+^-ATPase (A016-1-1) were measured using commercial kits (Nanjing Jiancheng Bioengineering Institute, Nanjing, China).

### 2.5. Data Processing

In the acute toxicity test group, mortality rates at 24, 48, 72, and 96 h were recorded across all concentration gradients. Using probit analysis, regression equations describing the relationship between mortality rates and experimental concentrations were derived. The modified Kou’s method was employed to calculate key toxicological parameters based on these regression models. to calculate the median lethal concentration (LC_50_) and 95% confidence interval (95% CI) [28].

Data in the physiological and biochemical group were expressed as the mean ± standard deviation (SD). A two-way analysis of variance (two-way ANOVA) was employed to evaluate the main effects and interaction effects of salinity (fixed factor) and exposure time (fixed factor) on physiological indices. When the indicated significant differences (*p* < 0.05), Tukey’s post hoc test was further used for pairwise comparisons among multiple groups to identify specific significant differences among salinity gradients, time points, or under salinity–time interactions. All statistical analyses were conducted using IBM SPSS Statistics 20.0, and data were plotted with GraphPad Prism 10.1.

### 2.6. Formatting of Mathematical Components

Equation (1) shows the median lethal concentration (LC_50_):

(1) $$Ig{LC}_{50}=X_{m}-d\left(\Sigma P_{i}-0.5\right)$$

where Xₘ is the logarithm of the highest concentration, d is the logarithmic difference between consecutive concentrations, Pᵢ represents the mortality rate.

Equation (2) is the 95% confidence interval (95% CI):

(2) $$\;{Ig}^{-1}\left(Ig{LC}_{50}\pm 1.96SD\right)$$

Equation (3) is the formula for the standard deviation (SD) used to measure the dispersion of results:

(3) $$SD=d\sqrt{\frac{{\left(\sum P_{i}-\sum P_{i}\right)}^{2}}{n}}$$

where d is the logarithmic difference between consecutive concentrations, Pᵢ represents the mortality rate, n denotes the sample size

Equation (4) Estimation of Safe Concentration (SC) via an empirical formula for acute fish toxicity tests:

(4) $$SC=48\;h\times 0.3/{24\;(h\;{LC}_{50}/48\;h\;{LC}_{50})}^{2}$$

where 48 h is a fixed time parameter, 0.3 an empirical coefficient, 24 h LC_50_/48 h LC_50_ test-derived concentrations inducing 50% fish mortality in 24/48 h, and SC the ecological safety threshold for fish under acute exposure.

## 3. Results

### 3.1. Behavioral Responses, Mortality, and Correlation Analysis Under Different Salinities

**Figure 2:**
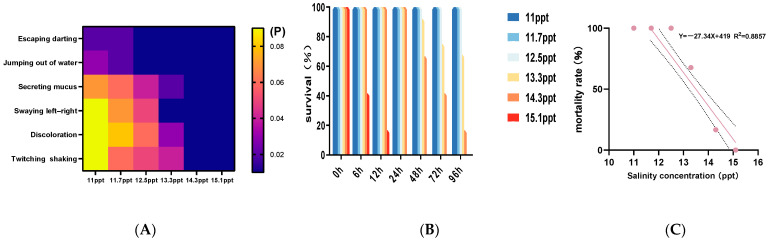
Behavioral responses, mortality and 96 h 95% confidence interval of *Triplophysa strauchii* under different salinities. (**A**) Heat map showing the response intensity P of phenotypic traits under salinity gradients. Rows represent the observed phenotypic traits (vertical axis: escape/darting, leaping out of water, mucus secretion, side-to-side swaying, discoloration, twitching/shaking), and columns represent salinity concentrations (horizontal axis, unit: ppt). The color scale indicates the magnitude of the response intensity (range: 0.02–0.08, arbitrary units). (**B**) Mortality rates at each time period under different salinities. (**C**) The relationship between salinity concentration (ppt) and mortality rate (%). The solid line represents the linear regression fit (Y = −27.34X + 419, R^2^ = 0.8857) the dashed lines indicate the 95% confidence intervals, and the pink dots represent the designed concentration values.

All experimental groups showed distinct behavioral changes under salinity stress, which worsened with higher salinity and longer exposure (Figure 2A). Initially, fish became restless, swam near the surface, circled the tank rapidly, leaped out of water, and beat their gill covers more frequently; later, they secreted more mucus (turning the water yellow), stayed motionless at the bottom, and reacted slowly to stimuli; eventually, mucus decreased, bodies turned pale, and red spots appeared on the snout, fin bases, and eyeballs, with the fish in 13.3–15.1 ppt groups swimming sideways with swaying and those in the 14.3–15.1 ppt groups showing severe terminal behaviors like thrashing, convulsions, weak gill movement, floating, and sinking belly-up. The control group remained normal with no deaths. Survival rates dropped as salinity and exposure time increased, showing toxic effects were linked to both factors (Figure 2B), and from mortality data, the 96-hour median lethal salinity (LC_50_) was 13.31 ppt. According to Equation (4), the safe concentration calculated is 4.04 ppt (Figure 2C).

### 3.2. Trends in Enzyme Activities of Four Tissues and Plasma Under Different Salinities

#### 3.2.1. Effects of Salinity Stress on Gill Osmoregulation and Antioxidation

**Figure 3:**
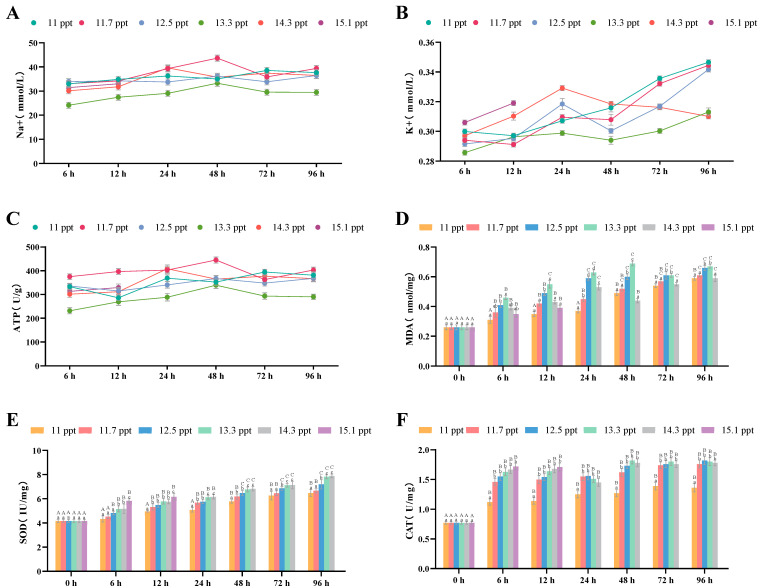
The effects of salinity stress on gill osmoregulation, antioxidant system and tissue morphology. (**A**–**C**): Na^+^ and K^+^ concentrations, ATP activity; (**D**–**F**): MDA content, SOD activity, CAT activity; different lowercase letters denote significant differences (*p* < 0.05) among different salinities at the same time point, while different uppercase letters indicate significant differences (*p* < 0.05) across different time points within the same salinity.

Following salt stress within 96 h, the Na^+^ concentration in the gill tissues of *Triplophysa strauchii* first increased significantly (*p* < 0.05) and then returned to normal levels (*p* > 0.05) (Figure 3A). Within 24 h, the K^+^ concentration in the gill tissues of all salinity groups rose significantly with prolonged salt stress (*p* < 0.05); in the 14.3 ppt group, it decreased after 24 h and returned to normal by 96 h (*p* > 0.05), while in other groups, it continued to increase throughout the stress period (*p* < 0.05) (Figure 3B). ATPase levels in each salinity group fluctuated but not significantly within 96 h (*p* > 0.05) (Figure 3C).

For antioxidant indices, gill MDA content in groups with salinity below 14.3 ppt increased significantly with higher salinity, but showed a significant downward trend when salinity was ≥14.3 ppt (*p* < 0.05) (Figure 3D). SOD (Figure 3E) and CAT (Figure 3F) contents rose significantly with increasing salinity (*p* < 0.05). Over 96 h, MDA, SOD, and CAT contents in all salinity groups increased significantly as stress time prolonged (*p* < 0.05).

#### 3.2.2. Effects of Salinity Stress on Kidney Osmoregulation and Antioxidation

**Figure 4:**
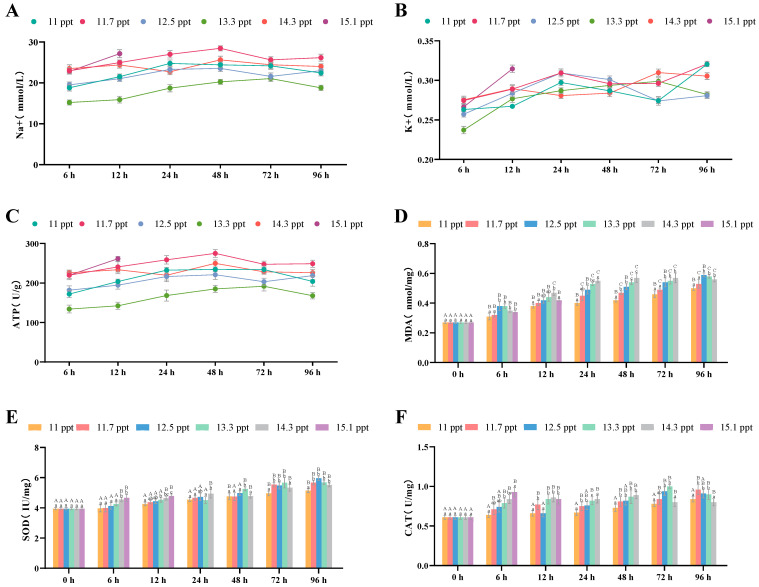
The effects of salinity stress on renal osmoregulation, antioxidant system and tissue morphology. (**A**–**C**): Na^+^ and K^+^ concentrations, ATP activity; (**D**–**F**): MDA content, SOD activity, CAT activity; different lowercase letters denote significant differences (*p* < 0.05) among different salinities at the same time point, while different uppercase letters indicate significant differences (*p* < 0.05) across different time points within the same salinity.

Within 96 h of salt stress, the Na^+^ concentration (Figure 4A) and ATPase level (Figure 4C) in the renal tissues of *Triplophysa strauchii* increased within 48 h and then decreased significantly after 48 h (*p* < 0.05). The K^+^ concentration in all salinity groups rose continuously over the 96-hour period (*p* < 0.05) (Figure 4B).

For antioxidant indices in renal tissues, MDA (Figure 4D), SOD (Figure 4E), and CAT (Figure 4F) contents all increased significantly with higher salinity (*p* < 0.05) and also rose significantly in all salinity groups as stress time extended within 96 h (*p* < 0.05).

#### 3.2.3. Effects of Salinity Stress on Liver Osmoregulation and Immune Function

**Figure 5:**
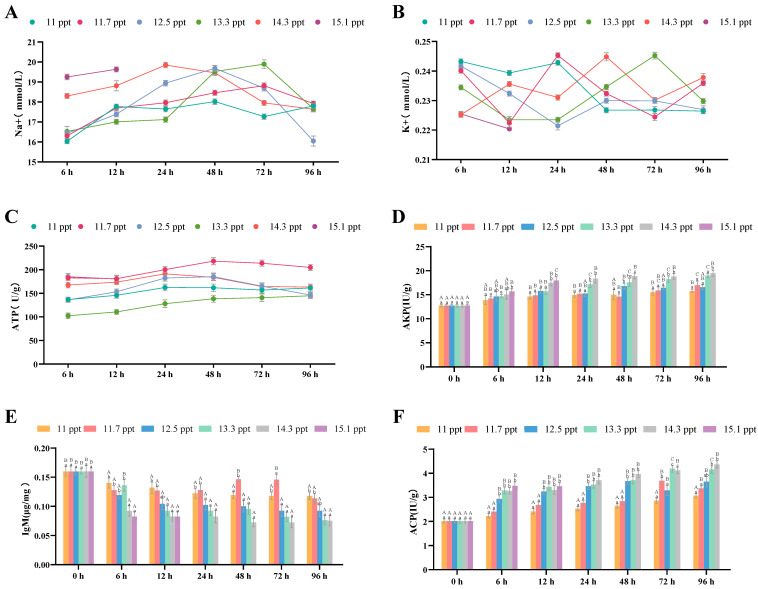
The effects of salinity stress on hepatic osmoregulation, immune enzymes and tissue morphology. (**A**–**C**): Na^+^ and K^+^ concentrations, ATP activity; (**D**–**F**): AKP activity, IgM content, ACP activity; different lowercase letters denote significant differences (*p* < 0.05) among different salinities at the same time point, while different uppercase letters indicate significant differences (*p* < 0.05) across different time points within the same salinity.

In the 11 ppt salinity group, the Na^+^ content in *Triplophysa strauchii* liver tissues showed no significant fluctuation with salt stress time, while in the 11.7 ppt, 12.5 ppt, and 14.3 ppt groups, Na^+^ content first increased and then decreased (Figure 5A). The K^+^ content in liver tissues of all salinity groups fluctuated irregularly with stress time (Figure 5B), and the ATPase content in each group showed no significant fluctuation with stress time (Figure 5C).

For immune indices in liver tissues, AKP (Figure 5D) and ACP (Figure 5F) contents increased significantly with higher salinity (*p* < 0.05), while IgM (Figure 5E) content decreased significantly with increasing salinity (*p* < 0.05). Over 96 h, AKP and ACP contents in all salinity groups rose significantly as stress time prolonged (*p* < 0.05), whereas IgM showed the opposite trend (*p* < 0.05).

#### 3.2.4. Effects of Salinity Stress on Intestinal Osmoregulation and Antioxidation

**Figure 6:**
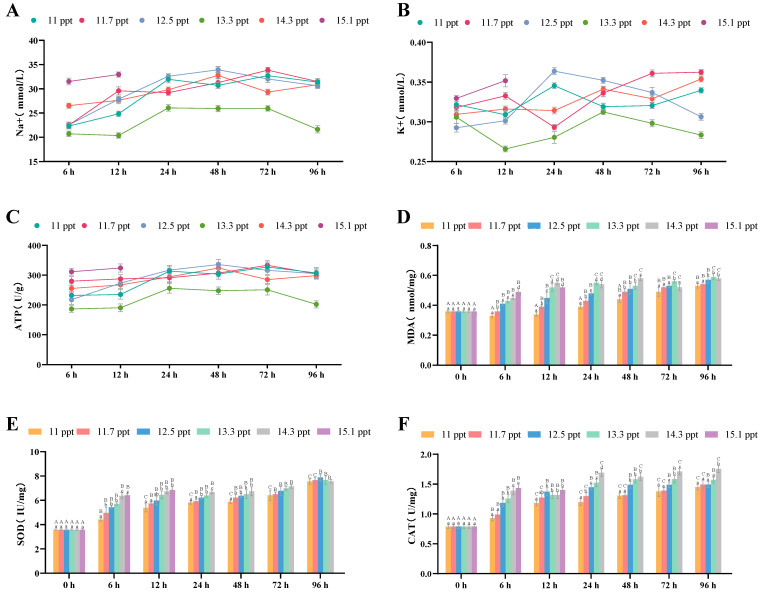
The effects of salinity stress on intestinal osmoregulation, antioxidant system and tissue morphology. (**A**–**C**): Na^+^ and K^+^ concentrations, ATP activity; (**D**–**F**): MDA content, SOD activity, CAT activity; different lowercase letters denote significant differences (*p* < 0.05) among different salinities at the same time point, while different uppercase letters indicate significant differences (*p* < 0.05) across different time points within the same salinity.

In the 11.7 ppt, 12.5 ppt, and 14.3 ppt groups, the Na^+^ content in the intestinal tissues of Triplophysa strauchii increased with salt stress time (Figure 6A). The K^+^ content in the intestinal tissues of all salinity groups fluctuated irregularly with stress time (Figure 6B), and the ATPase content in the intestinal tissues of each group showed no significant fluctuation with salt stress time (Figure 6C).

For antioxidant indices in intestinal tissues, MDA (Figure 6D), SOD (Figure 6E), and CAT (Figure 6F) contents all increased significantly with higher salinity (*p* < 0.05). Additionally, within 96 h, these three indices in all salinity groups rose significantly as stress time prolonged (*p* < 0.05).

#### 3.2.5. Effects of Salinity Stress on Plasma Osmoregulation and Antioxidation

**Figure 7:**
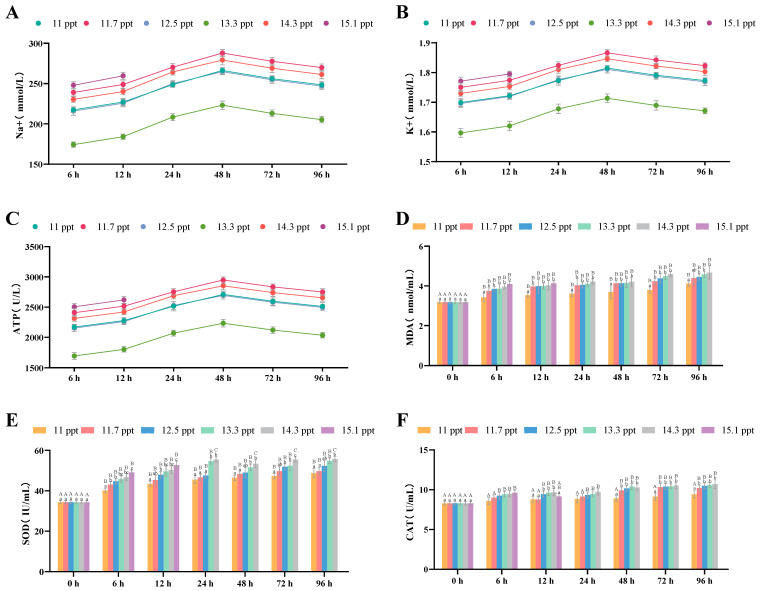
The effects of salinity stress on plasma osmoregulation and antioxidant system. (**A**–**C**): Na^+^ and K^+^ concentrations, ATP activity; (**D**–**F**): MDA content, SOD activity, CAT activity; different lowercase letters denote significant differences (*p* < 0.05) among different salinities at the same time point, while different uppercase letters indicate significant differences (*p* < 0.05) across different time points within the same salinity.

Within 96 h, the Na^+^ content (Figure 7A), K^+^ content (Figure 7B), and ATPase content (Figure 7C) in the plasma of *Triplophysa strauchii* across all salinity groups first increased and then decreased.

For plasma antioxidant indices, MDA (Figure 7D), SOD (Figure 7E), and CAT (Figure 7F) contents all showed a significant upward trend with increasing salinity (*p* < 0.05). Additionally, within 96 h, these three indices in all salinity groups significantly increased as stress time prolonged (*p* < 0.05).

## 4. Discussion

### 4.1. Effects of Salinity on Fish Behavior and Survival

Salinity represents a critical environmental stressor that promotes behavioral differentiation in fish. Acute salinity fluctuations trigger a hierarchical response cascade, ranging from active behavioral avoidance to physiological compensatory regulation [2,29,30]. In this study, *Triplophysa strauchii* exhibited restlessness and frequent leaping during the early stage of acute salinity stress (0–6 h), behaviors that share striking similarity with salinity avoidance responses observed in benthic fish such as European sea bass (*Dicentrarchus labrax*) [31], *Tarim schizothorax* [32], and *Iberian barbel* [29]. This behavior essentially aims to alleviate gill surface ionic imbalance by increasing water exchange frequency, reflecting the rapid sensory capability of benthic fish to local salinity fluctuations. *Triplophysa strauchii* has long inhabited slow-flowing bottom layers, relying on body surface mucus and gill epithelial cells to sense water ion changes. Through leaping behavior, it accelerates material exchange with the surrounding water, rapidly activating the internal salt regulation mechanism to cope with suddenly elevated salinity. However, at salinities ≥ 12.5 ppt, fish exhibited body fading and whitening. This occurs because the mucus layer, as the first line of defense against external ionic shock, undergoes mucin degradation, exacerbating the direct exposure of gill epithelium to hypertonic environments. This triggers excessive consumption of Na^+^/K^+^-ATPase and overloads the osmoregulatory system, leading to physiological disorders such as abnormal mucus secretion and color fading [5]. This phenomenon mirrors the mucosal barrier disruption mechanism in African catfish under high salinity stress, both demonstrating the damaging effect of hypertonic environments on the surface protective system of fish [33]. Subsequent symptoms such as lateral swimming, weakened gill cover respiration, and abdominal upward position occur because salinity disrupts the body’s osmotic pressure, leading to an osmotic imbalance and the inability to maintain normal swimming posture. This is consistent with the stress responses of most fish under high saline–alkali stress [2]. Toxicity effect analysis showed that the 96 h LC_50_ of *Triplophysa strauchii* to NaCl was 13.31 ppt, even exceeding that of some euryhaline freshwater fish such as *crucian carp* [34] and *Songpu mirror carp* [35]. This difference may be attributed to the high evaporation in the Emin River basin, its habitat, leading to large salinity variations and thus enhancing its tolerance.

### 4.2. The Impact of Salinity on Osmoregulation, Immune Response, and Oxidative Stress in Fish

As a critical environmental factor governing fish survival, salinity fluctuations exert multifaceted regulatory effects on fish physiological functions [2]. Such fluctuations not only modulate osmotic balance through organs like gills and kidneys, but also elicit stress responses in the antioxidant system to counteract reactive oxygen species (ROS) damage, while imposing significant impacts on the defensive capacities of the immune system [36].

#### 4.2.1. The Impact of Salinity on Osmoregulation in Fish

As a key osmoregulatory organ in direct contact with water, the gill actively extrudes excessive Na^+^ via Na^+^/K^+^-ATPase pumps on the surface of epithelial cells, while the kidney maintains ionic balance by reabsorbing K^+^ in renal tubules [37,38,39]. In *Pelteobagrus fulvidraco*, hypoosmotic stress induces vasodilation in gill filaments and decreased spacing between gill lamellae, synergistically improving ion exchange efficiency and gas exchange capacity [40]. In juvenile *Mugil cephalus*, salinity stress significantly increases Na^+^/K^+^-ATPase activity in gill filaments, accompanied by epithelial cell hyperplasia in gill lamellae to enhance ion transport capacity [41]; for *Oncorhynchus mykiss,* under acute salinity conditions, the kidney maintains intracellular K^+^ homeostasis via active transport, Reduce Na^+^ loss in urine [42].This indicates that both freshwater and marine fish commonly develop primary osmoregulatory mechanisms through physiological responses in gills and kidneys when facing salinity changes [43]. In this study, ATPase activity in the gill tissues of *Triplophysa xinjiangensis* significantly increased within 6 h of salinity stress (Figure 3C), with concurrent elevation in renal tissues (Figure 4C). By regulating ion transport capacity (Figure 3A,B and Figure 4A,B), these findings demonstrate that gills and kidneys exhibit high sensitivity to external salinity, confirming their roles as core osmoregulatory organs. In contrast, the liver–intestine system, primarily responsible for energy metabolism and nutrient absorption, showed delayed responses to salinity stress, with significant fluctuations observed only after 12 h (Figure 5C and Figure 6C). This pattern aligns with observations in *Paramisgurnus dabryanus* [44] and *Mastacembelus sinensis* [45,46], where gill and renal tissues completed enzymatic activity peaks within 48 h, while intestinal ATPase activity fluctuated at later stages of salinity stress. These results further indicate that gills and kidneys serve as the main regulatory organs in *Triplophysa strauchii*, whereas the liver–intestine system exhibited osmotic regulatory lag, consistent with the metabolic organ response delay theory.

Plasma Na^+^/K^+^-ATPase activity in *Triplophysa strauchii* showed a continuous increase with rising salinity, and Na^+^/K^+^ concentrations were significantly positively correlated with enzymatic activity (r = 0.89, *p* < 0.01). This phenomenon aligns with the ecological habits of benthic fish such as *Monopterus albus* [47], which frequently encounter local salinity pulses in their benthic lifestyle. In contrast to the euryhaline fish *Oreochromis* spp., which maintains plasma ionic homeostasis through active regulation, *Triplophysa* tends to achieve short-term adaptation via ionic conformity regulation [48]. This strategy reflects the distinct response mechanism of *Triplophysa* to salinity fluctuations in arid-region inland rivers. Notably, in *Triplophysa strauchii*, gill tissue Na^+^/K^+^-ATPase activity increased at salinities ≤ 13.3 ppt, accompanied by active Na^+^ extrusion via ion transport. This phenomenon indicates that *Triplophysa strauchii* still exhibits limited active regulatory capacity. Meanwhile, plasma K^+^ concentration increased concurrently (Figure 7B), further demonstrating the coordinated transport mechanism of Na^+^/K^+^-ATPase (3Na^+^ efflux/2K^+^ influx).

#### 4.2.2. The Impact of Salinity on the Antioxidant System in Fish

When fish encounter salinity stress, excessive intracellular reactive oxygen species (ROS) induce oxidative damage to biological macromolecules such as proteins and lipids. As key enzymes of the antioxidant defense system, superoxide dismutase (SOD) and catalase (CAT) maintain the redox balance by catalyzing ROS into H_2_O_2_ (via SOD) and further decomposing H_2_O_2_ into H_2_O and O_2_ (via CAT) [49,50]. In this study, SOD and CAT activities in gill tissues under salinities ≤ 13.3 ppt exhibited a pattern of “rapid increase followed by slow decrease” peaking at 96 h (Figure 3E,F). Conversely, renal tissue activities showed continuous fluctuating increases with rising salinity (Figure 4E,F). This pattern is consistent with the “increase-then-decrease” trend observed in SOD and CAT activities of gill, intestine, and liver tissues in Cyprinus carpio [51]. This study confirmed a universal “initial increase followed by decrease” pattern in antioxidant enzyme activities of fish under salinity stress. In *Triplophysa strauchii*, intestinal enzyme activities were rapidly activated at 6 h, then remained stable with a slight decline under salinities ≥ 14.3 ppt (Figure 6E,F). However, MDA levels showed a continuous increase (Figure 6D), indicating that salinity approached and exceeded the physiological tolerance threshold, leading to ROS production rates surpassing the enzymatic catalysis capacity. For example, in loach (*Misgurnus anguillicaudatus*) subjected to 12 ppt salinity stress for 96 h, intestinal SOD and CAT activities decreased compared to the control group, while MDA levels increased [52]. These findings are highly consistent with the antioxidant enzyme inactivation and oxidative damage characteristics observed in the intestinal tissues of *Triplophysa strauchii* under salinities ≥ 14.3 ppt (Figure 6D–F). Thus, the physiological tolerance threshold of *Triplophysa strauchii* is <14.3 ppt. Once this threshold is exceeded, excessive ROS attack tissues and organs, leading to lipid peroxidation of gill filament epithelium, compromised gill gas exchange, reduced renal excretory capacity, and impaired intestinal absorption. This also induces inflammatory cell infiltration in liver tissue, thereby exacerbating tissue damage.

#### 4.2.3. The Impact of Salinity on Fish Immune System

Salinity can exert complex effects on fish immune responses via pathways such as osmoregulation and oxidative stress. As a key indicator of fish humoral immunity, IgM reflects the specific defense capacity against pathogens. Abrupt salinity decrease or increase triggers stress responses in fish, inhibiting IgM synthesis [53]. As lysosomal enzymes, alkaline phosphatase (AKP), and acid phosphatase (ACP) serve dual functions of immune defense and metabolic regulation. Their activities are associated with tissue damage or immune cell activation, fluctuating significantly under salinity-induced osmotic imbalance or immune stress [54]. They primarily participate in immune responses by catalyzing the hydrolysis of phosphate esters to generate inorganic phosphate and promote ATP synthesis, with their activity serving as an indicator of organismal immune capacity [55]. In this study, the activities of AKP and ACP in the liver tissues of *Triplophysa* showed a typical “increase-then-decrease” fluctuation with rising salinity (Figure 5D,F). AKP activity peaked in the 13.3 ppt salinity group and then significantly decreased in the 15.1 ppt group (Figure 5D), whereas ACP activity reached its maximum in the 11.7 ppt group and was lowest under the high-salinity condition of 15.1 ppt (Figure 5F). This pattern aligns with findings in juvenile Acipenser sinensis, where hepatic ACP activity continuously increased with salinity up to 20 ppt but decreased significantly beyond this threshold [56]. In a gradual salinity stress experiment with channel catfish (*Ictalurus punctatus*), hepatic AKP activity peaked at 12 ppt, decreasing at salinities ≥ 16 ppt. ACP activity, conversely, increased significantly at 10 ppt and declined at 20 ppt [11]. In conjunction with the findings of this study on *Triplophysa strauchii*—where AKP/ACP activity thresholds (11.7–13.3 ppt) coincided with the species’ natural salinity tolerance—these results further validate that immune enzyme activity thresholds act as dynamic physiological boundary markers for fish salinity niches. This provides mechanistic evidence for the “osmo-immune trade-off” hypothesis, suggesting that enzyme activity decline beyond thresholds may serve as an early warning signal for ecological niche restriction.

In summary, long-term exposure to high salinity may lead to abnormal growth hormone secretion and impaired IgM synthesis in juvenile *Triplophysa strauchii* via oxidative stress, thereby increasing the risk of infection by pathogens such as Aeromonas *hydrophila*. Notably, the safe concentration determined in this study (4.05 ppt) significantly differs from the background salinity of *Triplophysa* habitats, indicating that even sub-lethal salinity levels may cause a population decline by inhibiting IgM synthesis and reducing juvenile survival rates through chronic stress.

## 5. Conclusions

This study investigated the physiological responses and tolerance mechanisms of *Triplophysa strauchii* to acute salinity stress. The key findings are as follows: first, the 96-hour median lethal concentration (LC_50_) of *Triplophysa strauchii* to salinity was determined to be 13.31 ppt, with a safe survival concentration (SC) of 4.05 ppt, providing critical baseline data for assessing its survival risk in salinizing habitats. Second, gill and kidney tissues exhibited high sensitivity to salinity fluctuations, rapidly regulating Na^+^/K^+^-ATPase activity within 6–24 h to maintain osmotic balance, confirming their role as primary osmoregulatory organs. In contrast, liver and intestinal tissues showed delayed responses, with significant enzymatic changes observed only after 12 h, indicating a secondary role in osmoregulation. Third, under short-term salinity ≤ 13.3 ppt, *Triplophysa strauchii* maintained homeostasis through adaptive adjustments in antioxidant (SOD, CAT) and immune (IgM, ACP, AKP) systems, with reversible physiological responses. However, salinity ≥ 14.3 ppt triggered irreversible oxidative stress overload, and immune system collapse, threatening survival (Table 1). These results reveal the salinity tolerance threshold and physiological damage mechanisms of *Triplophysa strauchii*, highlighting the vulnerability of this species to salinization. Further research on chronic salinity exposure and wild population validation is needed to comprehensively understand its adaptation strategies, which will support conservation efforts for this “Near Threatened” indicator species in arid inland basins.

## Figures and Tables

**Table 1:** Summary of key indicator trends in *Triplophysa strauchii* tissues under salinity stress.

Index Category	Specific Index	Tissue	Trend with Increasing Salinity	Trend with Prolonged Stress (0–96 h)	Key Time Points
Ion Regulation	Na^+^ Concentration	Gill	Low-salinity groups (≤13.3 ppt): first increase then recovery; high-salinity groups (≥14.3 ppt): persistently high	Peaks at 24–48 h; low-salinity groups recover after 72 h	24–48 h
	K^+^ Concentration	Gill	Overall increase; 14.3 ppt group decreases after 24 h	Most groups show continuous increase; 14.3 ppt group decreases after 24 h	Most groups show continuous increase; 14.3 ppt group decreases after 24 h
	Na^+^/K^+^-ATPase Activity	Gill	No significant regular fluctuation within 96 h	No significant regular fluctuation	48 h (turning point)
	Na^+^ Concentration	Kidney	increase	Increases by 48 h, then decreases significantly after 48 h	48 h (peak)
	K^+^ Concentration	Kidney	Continuous increase	Continuous increase	96 h (highest value)
	Na^+^/K^+^-ATPase Activity	Kidney	11 ppt group: no significant fluctuation; 11.7, 12.5, 14.3 ppt groups: first increase then decrease	Increases by 48 h, then decreases significantly after 48 h	48 h (peak)
	Na^+^ Concentration	Liver	increase	trend of increasing first and then decreasing	48 h (turning point)
	K^+^ Concentration	Liver	Irregular fluctuations over time	Irregular fluctuations over time	/
	Na^+^/K^+^-ATPase Activity	Liver	No significant fluctuation	No significant fluctuation	/
	Na^+^ Concentration	Intestine	11.7, 12.5, 14.3 ppt groups: increase with stress time	Increase with stress time	96 h (highest value)
	K^+^ Concentration	Intestine	Irregular fluctuations over time	Irregular fluctuations over time	24 h (turning point)
	Na^+^/K^+^-ATPase Activity	Intestine	No significant fluctuation	No significant fluctuation	/
	Na^+^ Concentration	Plasma	increase	First increase then decrease	48 h (peak)
	K^+^ Concentration	Plasma	increase	First increase then decrease	48 h (peak)
	Na^+^/K^+^-ATPase Activity	Plasma	increase	First increase then decrease	48 h (peak)
Antioxidant System	MDA Content	Gill	<14.3 ppt: increase; ≥14.3 ppt: decrease	Continuous increase	96 h (highest value)
	SOD Activity	Gill	Significant increase	Continuous significant increase	96 h (highest value)
	CAT Activity	Gill	Significant increase	Continuous increase	96 h (highest value)
	MDA Content	Kidney	Significant increase	Continuous significant increase	96 h (highest value)
	SOD Activity	Kidney	Significant increase	Continuous increase	96 h (highest value)
	CAT Activity	Kidney	Significant increase	Continuous significant increase	96 h (highest value)
	MDA Content	Intestine	Significant increase	Continuous increase	96 h (highest value)
	SOD Activity	Intestine	Significant increase	Continuous significant increase	96 h (highest value)
	CAT Activity	Intestine	Significant increase	Continuous increase	96 h (highest value)
	MDA Content	Plasma	Significant increase	Continuous significant increase	96 h (highest value)
	SOD Activity	Plasma	Significant increase	Continuous increase	96 h (highest value)
	CAT Activity	Plasma	Significant increase	Continuous significant increase	96 h (highest value)
Immune Indicators	AKP Content	Liver	Significant increase	Continuous significant increase	96 h (highest value)
	IgM Content	Liver	Significant decrease	Continuous decrease	96 h (lowest value)
	ACP Content	Liver	Significant increase	Continuous significant increase	96 h (highest value)

## Data Availability

The raw data supporting the conclusions of this article will be made available by the authors on request.

## Abbreviations

The following abbreviations are used in this manuscript:

SOD	Superoxide dismutase
CAT	Catalase
MDA	Malondialdehyde
IgM	Immunoglobulin M
AKP	Alkaline phosphatase
ACP	Acid phosphatase
ROS	Reactive oxygen species

## References

[1] Korus J., Filgueira R., Grant J. (2024). Influence of temperature on the behaviour and physiology of Atlantic salmon (*Salmo Salar*) on a commercial farm. Aquaculture.

[2] Kültz D. (2015). Physiological mechanisms used by fish to cope with salinity stress. J. Exp. Biol..

[3] Sundby S., Kristiansen T. (2015). The principles of buoyancy in marine fish eggs and their vertical distributions across the world oceans. PLoS ONE.

[4] Hieu D.Q., Hang B.T.B., Lokesh J., Garigliany M.M., Huong D.T.T., Yen D.T., Liem P.T., Tam B.M., Hai D.M., Son V.N. (2022). Salinity significantly affects intestinal microbiota and gene expression in striped catfish juveniles. Appl. Microbiol. Biotechnol..

[5] Alford S.B., Husband S.A., Martin C.W. (2024). Resilience to changes in salinity and predator diversity in representative Gulf of Mexico estuarine fish predator-prey interactions. J. Exp. Mar. Biol. Ecol..

[6] Ma Q., Liu X., Feng W., Liu S., Zhuang Z. (2018). Analyses of the molecular mechanisms associated with salinity adaption of Trachidermus fasciatus through combined iTRAQ-based proteomics and RNA sequencing-based transcriptomics. Prog. Biophys. Mol. Biol..

[7] Greenwell M.G., Sherrill J., Clayton L.A. (2003). Osmoregulation in fish. Mechanisms and clinical implications. Vet. Clin. N. Am. Exot. Anim. Pract..

[8] Maraschi A.C., Faria S.C., McNamara J.C. (2021). Salt transport by the gill Na^+^-K^+^-2Cl^−^ symporter in palaemonid shrimps: Exploring physiological, molecular and evolutionary landscapes. Comp. Biochem. Physiol. Part A Mol. Integr. Physiol..

[9] Avella M., Ducoudret O., Pisani D.F., Poujeol P. (2009). Swelling-activated transport of taurine in cultured gill cells of sea bass: Physiological adaptation and pavement cell plasticity. Am. J. Physiol. Regul. Integr. Comp. Physiol..

[10] Resende A.D., Lobo-da-Cunha A., Malhão F., Franquinho F., Monteiro R.A., Rocha E. (2010). Histological and stereological characterization of brown trout (*Salmo trutta* f. *fario*) trunk kidney. Microsc. Microanal..

[11] Tang L., Duan Y., Xie B., Liu H., Zhong L., Wang M., Liu J., Su C., Chen X., Zhang S. (2025). Effects of salinity stress on the growth performance, histological characteristics, and expression of genes related to apoptosis and immunity in channel catfish (*Ictalurus punctatus*). J. Fish Biol..

[12] Thebault M.-T., Raffin J.-P. (1984). Properties of the lysosomes from liver and gill of rainbow trout, Salmo gairdnerii R.: Effect of starvation, salinity and 2, 4, 5-T. Comp. Biochem. Physiol. Part B Comp. Biochem..

[13] Lee D.-W., Choi Y.-U., Park H.-S., Park Y.-S., Choi C.Y. (2022). Effect of low pH and salinity conditions on the antioxidant response and hepatocyte damage in juvenile olive flounder Paralichthys olivaceus. Mar. Environ. Res..

[14] Sundell K.S., Sundh H. (2012). Intestinal fluid absorption in anadromous salmonids: Importance of tight junctions and aquaporins. Front. Physiol..

[15] Chourasia T.K., D’Cotta H., Baroiller J.F., Slosman T., Cnaani A. (2018). Effects of the acclimation to high salinity on intestinal ion and peptide transporters in two tilapia species that differ in their salinity tolerance. Comp. Biochem. Physiol. A Mol. Integr. Physiol..

[16] Banh S., Wiens L., Sotiri E., Treberg J.R. (2016). Mitochondrial reactive oxygen species production by fish muscle mitochondria: Potential role in acute heat-induced oxidative stress. Comp. Biochem. Physiol. B Biochem. Mol. Biol..

[17] Shivakumar K., Jayaraman J. (1984). Salinity adaptation in fish: Effect of thyroxine on mitochondrial status. Arch. Biochem. Biophys..

[18] Vahdatiraad L., Heidari B., Zarei S., Sohrabi T., Ghafouri H. (2023). Biological responses of stellate sturgeon fingerlings (*Acipenser stellatus*) immersed in HSP inducer to salinity changes. Mar. Environ. Res..

[19] Schmitz M., Ziv T., Admon A., Baekelandt S., Mandiki S.N.M., L’Hoir M., Kestemont P. (2017). Salinity stress, enhancing basal and induced immune responses in striped catfish *Pangasianodon hypophthalmus* (Sauvage). J. Proteom..

[20] Aiyixia X., Xin J. (2002). Hydrological Characteristics of the Emin River Basin. J. China Hydrol..

[21] Gao J., Jing L., Li H., Miao Y., Jing L. (2024). Variation Characteristics of Precipitation and Meteorological Drought in Xinjiang Tacheng Prefecture during Recent 60 Years. Meteorol. Res. Plateau Mt. Areas.

[22] Chen H.T., Tang Y. (2010). Variation Characteristics of Precipitation in Xinjiang Tacheng Prefecture during Recent 55 Years. J. Water Resour. Water Eng..

[23] Jiang Z., Jiang W. (2015). Analysis of Social Benefits and Ecological Effects from Comprehensive Development of Emin River Basin. Jilin Water Resour. Hydropower.

[24] Zongxia W., Suxia L. (2023). Estimation of Groundwater Storage and Its Spatiotemporal Evolution Patterns in the Northern Slope of Tian Shan during 1990–2020. Acta Geogr. Sin..

[25] Aynu R.L.Z.H.Z. (2010). Impacts of Climate Change on Hydrological Regime in Baiyang River Basin, Tacheng, Xinjiang during 1962–2007. J. Glaciol. Geocryol..

[26] Jiang Z., Jiang J., Wang Y., Zhang E., Zhang Y., Li L., Xie F., Cai B., Cao L., Zheng G. (2016). Red List of Vertebrates in China. Biodiversity.

[27] Wang Y., Chen P., Cui R., Si W., Zhang Y., Ji W. (2009). Heavy metal concentrations in water, sediment, and tissues of two fish species (*Triplohysa pappenheimi*, *Gobio hwanghensis*) from the Lanzhou section of the Yellow River, China. Environ. Monit. Assess..

[28] Li C., Wu M., Wong H. (2012). Comparison of Three Calculation Methods for Median Lethal Concentration (LC_50_). Prog. Vet. Med..

[29] Leite T., Branco P., Ferreira M.T., Santos J.M. (2022). Activity, boldness and schooling in freshwater fish are affected by river salinization. Sci. Total Environ..

[30] Bertolini F., Rohtla M., Parzanini C., Tomkiewicz J., Durif C.M.F. (2022). Blood-based gene expression as non-lethal tool for inferring salinity-habitat history of European eel (*Anguilla anguilla*). Sci. Rep..

[31] Islam M.J., Slater M.J., Kunzmann A. (2020). What metabolic, osmotic and molecular stress responses tell us about extreme ambient heatwave impacts in fish at low salinities: The case of European seabass, Dicentrarchus labrax. Sci. Total. Environ..

[32] Zhao H., Zhao H., Qiang Z., Nie Z., Wei J., Shen J. (2021). Effects of Photoperiod and Light Intensity on Survival, Feeding, Growth, and Saline-Alkali Tolerance in Juveniles of Tarim Schizothorax. South China Fish. Sci..

[33] Dawood M.A.O., Noreldin A.E., Sewilam H. (2022). Blood biochemical variables, antioxidative status, and histological features of intestinal, gill, and liver tissues of African catfish (*Clarias gariepinus*) exposed to high salinity and high-temperature stress. Environ. Sci. Pollut. Res. Int..

[34] Zheng W., Zhang Z., Zhang M. (2004). Study on Salinity and Alkalinity Tolerance in Juvenile Pengze Crucian Carp (*Carassius auratus* var. Pengze). J. Jimei Univ. Nat. Sci. Ed..

[35] Xu M., Wang S., Geng L., Jiang H., Cao D., Li C., Xu W. (2015). Effect of NaCl Salinity and NaHCO3 Alkalinity on Hatching and Larval Survival of Songpu Mirror Carp (*Cyprinus carpio* var. Songpu) and Fangzheng Crucian Carp (*Carassius auratus gibelio*). Chin. J. Zool..

[36] Wang X., Yao Q., Zhang D.-m., Lei X.-y., Wang S., Wan J.-w., Liu H.-j., Chen Y.-k., Zhao Y.-l., Wang G.-q. (2022). Effects of acute salinity stress on osmoregulation, antioxidant capacity and physiological metabolism of female Chinese mitten crabs (*Eriocheir sinensis*). Aquaculture.

[37] Sackville M.A., Cameron C.B., Gillis J.A., Brauner C.J. (2022). Ion regulation at gills precedes gas exchange and the origin of vertebrates. Nature.

[38] Prunet P., Bornancin M. (1989). Physiology of salinity tolerance in tilapia: An update of basic and applied aspects. Aquat. Living Resour..

[39] Edwards S.L., Marshall W.S., McCormick S.D., Farrell A.P., Brauner C.J. (2012). Principles and Patterns of Osmoregulation and Euryhalinity in Fishes. Fish Physiology.

[40] Tao Y.F., Qiang J., Dagoudo M., Zhu H.J., Bao J.W., Ma J.L., Li M.X., Xu P. (2021). Transcriptome profiling reveals differential expression of immune-related genes in gills of hybrid yellow catfish (*Tachysurus fulvidraco* ♀ × *Pseudobagrus vachellii* ♂) under hypoxic stress: Potential NLR-mediated immune response. Fish Shellfish Immunol..

[41] FE H. (1979). The gill arch of the mullet, Mugil cephalus. II. Modifigation in surface ultrastructure and Na, K-ATPase content during adaptation to various salinities. J. Exp. Zool..

[42] Al-Jandal N.J., Wilson R.W. (2011). A comparison of osmoregulatory responses in plasma and tissues of rainbow trout (*Oncorhynchus mykiss*) following acute salinity challenges. Comp. Biochem. Physiol. A Mol. Integr. Physiol..

[43] Geng L., Tong G., Jiang H., Xu W. (2016). Effect of salinity and alkalinity on luciobarbus capito Gill Na^+^/K^+^-ATPase enzyme activity, plasma ion concentration, and osmotic pressure. BioMed Res. Int..

[44] Liu Y., Wang Z. (2023). Ontogenetic Development of Gill and Na^+^/K^+^ ATPase in the Air-Breathing Loach. Fishes.

[45] Chu T.J., Jin Z., Liu K. (2021). The mitochondrial genome of Sinobdella sinensis (Synbranchiformes: Mastacembelidae) from China’s Qiantang River. Mitochondrial DNA B.

[46] Evans T.G., Kültz D. (2020). The cellular stress response in fish exposed to salinity fluctuations. J. Exp. Zool. A Ecol. Integr. Physiol..

[47] Zhou W., Song X., Chen G. (2014). Salinity and alkalinity tolerance of Monopterus albus. Freshw. Fish. Sci..

[48] Zidan E.M., Goma A.A., Tohamy H.G., Soliman M.M., Shukry M. (2022). Insight study on the impact of different salinity levels on behavioural responses, biochemical stress parameters and growth performance of African catfish (*Clarias gariepinus*). Aquac. Res..

[49] Jiang Y., Yuan C., Qi M., Liu Q., Hu Z. (2022). The Effect of Salinity Stress on Enzyme Activities, Histology, and Transcriptome of Silver Carp (*Hypophthalmichthys molitrix*). Biology.

[50] Li P., Liu W., Lu W., Wang J. (2022). Biochemical indices, gene expression, and SNPs associated with salinity adaptation in juvenile chum salmon (*Oncorhynchus keta*) as determined by comparative transcriptome analysis. PeerJ.

[51] Dawood M.A.O., Alkafafy M., Sewilam H. (2022). The antioxidant responses of gills, intestines and livers and blood immunity of common carp (*Cyprinus carpio*) exposed to salinity and temperature stressors. Fish Physiol. Biochem..

[52] Wang M., Xu W., Zou J., Li S., Song Z., Zheng F., Ji W., Xu Z., Wang Q. (2021). The Programming of Antioxidant Capacity, Immunity, and Lipid Metabolism in Dojo Loach (*Misgurnus anguillicaudatus*) Larvae Linked to Sodium Chloride and Hydrogen Peroxide Pre-treatment During Egg Hatching. Front. Physiol..

[53] van Muilekom D.R., Mueller J., Lindemeyer J., Schultheiß T., Maser E., Seibel H., Rebl A., Schulz C., Goldammer T. (2024). Salinity change evokes stress and immune responses in Atlantic salmon with microalgae showing limited potential for dietary mitigation. Front. Physiol..

[54] Wang Y., Li H., Wei J., Hong K., Zhou Q., Liu X., Hong X., Li W., Liu C., Zhu X. (2023). Multi-Effects of Acute Salinity Stress on Osmoregulation, Physiological Metabolism, Antioxidant Capacity, Immunity, and Apoptosis in Macrobrachium rosenbergii. Antioxidants.

[55] Bates J.M., Akerlund J., Mittge E., Guillemin K. (2007). Intestinal Alkaline Phosphatase Detoxifies Lipopolysaccharide and Prevents Inflammation in Zebrafish in Response to the Gut Microbiota. Cell Host Microbe.

[56] Zhao F., Qu L., Zhuang P., Zhang L., Liu J., Zhang T. (2011). Salinity tolerance as well as osmotic and ionic regulation in juvenile Chinese sturgeon (*Acipenser sinensis* Gray, 1835) exposed to different salinities. J. Appl. Ichthyol..

